# Centrosomes and associated proteins in pathogenesis and treatment of breast cancer

**DOI:** 10.3389/fonc.2024.1370565

**Published:** 2024-03-28

**Authors:** Harjot Athwal, Arpitha Kochiyanil, Vasudeva Bhat, Alison L. Allan, Armen Parsyan

**Affiliations:** ^1^ Department of Anatomy and Cell Biology, Schulich School of Medicine and Dentistry, Western University, London, ON, Canada; ^2^ Faculty of Science, Schulich School of Medicine and Dentistry, Western University, London, ON, Canada; ^3^ London Regional Cancer Program, London Health Sciences Centre, Lawson Health Research Institute, London, ON, Canada; ^4^ Department of Oncology, Schulich School of Medicine and Dentistry, Western University, London, ON, Canada; ^5^ Division of General Surgery, Department of Surgery, Schulich School of Medicine and Dentistry, Western University, London, ON, Canada; ^6^ Department of Surgery, St. Joseph’s Health Care London and London Health Sciences Centre, London, ON, Canada

**Keywords:** breast cancer, centrosome, centriole, PLK, AURK, CDK, CHK1/WEE1, CEP

## Abstract

Breast cancer is the most prevalent malignancy among women worldwide. Despite significant advances in treatment, it remains one of the leading causes of female mortality. The inability to effectively treat advanced and/or treatment-resistant breast cancer demonstrates the need to develop novel treatment strategies and targeted therapies. Centrosomes and their associated proteins have been shown to play key roles in the pathogenesis of breast cancer and thus represent promising targets for drug and biomarker development. Centrosomes are fundamental cellular structures in the mammalian cell that are responsible for error-free execution of cell division. Centrosome amplification and aberrant expression of its associated proteins such as Polo-like kinases (PLKs), Aurora kinases (AURKs) and Cyclin-dependent kinases (CDKs) have been observed in various cancers, including breast cancer. These aberrations in breast cancer are thought to cause improper chromosomal segregation during mitosis, leading to chromosomal instability and uncontrolled cell division, allowing cancer cells to acquire new genetic changes that result in evasion of cell death and the promotion of tumor formation. Various chemical compounds developed against PLKs and AURKs have shown meaningful antitumorigenic effects in breast cancer cells *in vitro* and *in vivo*. The mechanism of action of these inhibitors is likely related to exacerbation of numerical genomic instability, such as aneuploidy or polyploidy. Furthermore, growing evidence demonstrates enhanced antitumorigenic effects when inhibitors specific to centrosome-associated proteins are used in combination with either radiation or chemotherapy drugs in breast cancer. This review focuses on the current knowledge regarding the roles of centrosome and centrosome-associated proteins in breast cancer pathogenesis and their utility as novel targets for breast cancer treatment.

## Introduction

Breast cancer is a commonly diagnosed malignancy and one of the leading causes of cancer-related deaths among women worldwide (1). Current treatment strategies for breast cancer are based on the molecular subtype classification that takes into account cancer cell expression of hormone (estrogen and progesterone) receptors and the human epidermal growth factor 2 receptor (HER2) (2). Treatment modalities include surgery and/or radio-, chemo-, anti-endocrine, targeted and immune therapies. Despite advances in treatment options, the recurrence of breast cancer, chemoresistance, radioresistance and metastatic disease remain major areas of concern in achieving desired patient outcomes (3–5). Therefore, the development of novel drugs and therapies for breast cancer is of utmost clinical importance. Recent efforts in targeted drug development in oncology have been focused on inhibitors of cell cycle regulation. Aberrant regulation of cell cycle events is often observed in cancer cells (2, 6) and has been associated with breast cancer pathogenesis and the development of treatment resistance. This has led to the investigation of novel targets and the development of therapeutic agents targeting the cell cycle (2, 6–9). A large body of recent research indicates that centrosomes and their associated proteins play an important role in cell cycle progression and are thus highly promising targets for drug development in breast cancer (10–13). Here, we aim to review current knowledge on the role of centrosomes in breast cancer pathogenesis and highlight the development of novel drugs that target this critical hub of the cell cycle.

## Centrosomes and their associated proteins during the cell cycle

The mammalian cell cycle is an intricately coordinated and controlled process that ensures the proper division of cells (6) resulting in efficient segregation of genetic information. It is regulated by the activation and deactivation of various proteins throughout the five different stages of the cell cycle, including G0 (gap 0), G1, S (synthesis), G2 and M (mitosis) (6). The first four stages are known as interphase, the process by which cells grow and duplicate their genetic material to prepare for cell division (14). In the G0 phase, cells are quiescent and remain in a state of rest (14). Upon external stimulation, cells can enter G1 and produce mRNA and proteins needed for DNA replication in the S phase (14). Cells in G2 then ensure DNA replication is accurate and synthesize enzymes required for cell division (10, 14). Cells then proceed to divide during the M phase, which progresses through five main stages: prophase, metaphase, anaphase, telophase and cytokinesis (14). In prophase, the chromosomes condense, and the nuclear membrane starts to break down (15). The latter is a central step for assembling the mitotic spindle, consisting of the centrosome and microtubules (15). The microtubules of the developing spindle then attach to the kinetochore of chromosomes and pull them in opposite directions, thereby having a bi-directional orientation by the end of prophase (15). In metaphase, chromosomes are aligned at the equatorial plate and ensure the correct attachment of microtubules to the kinetochore, commonly referred to as the spindle assembly checkpoint (10, 16). Cells can then proceed to anaphase where sister chromatids separate to the spindle poles (16), followed by telophase where chromosomes decondense and the nuclear membrane reforms (16). As the last and final stage, the cytoplasm and chromosomes are equally divided into two daughter cells during cytokinesis (15).

**Figure 1:**
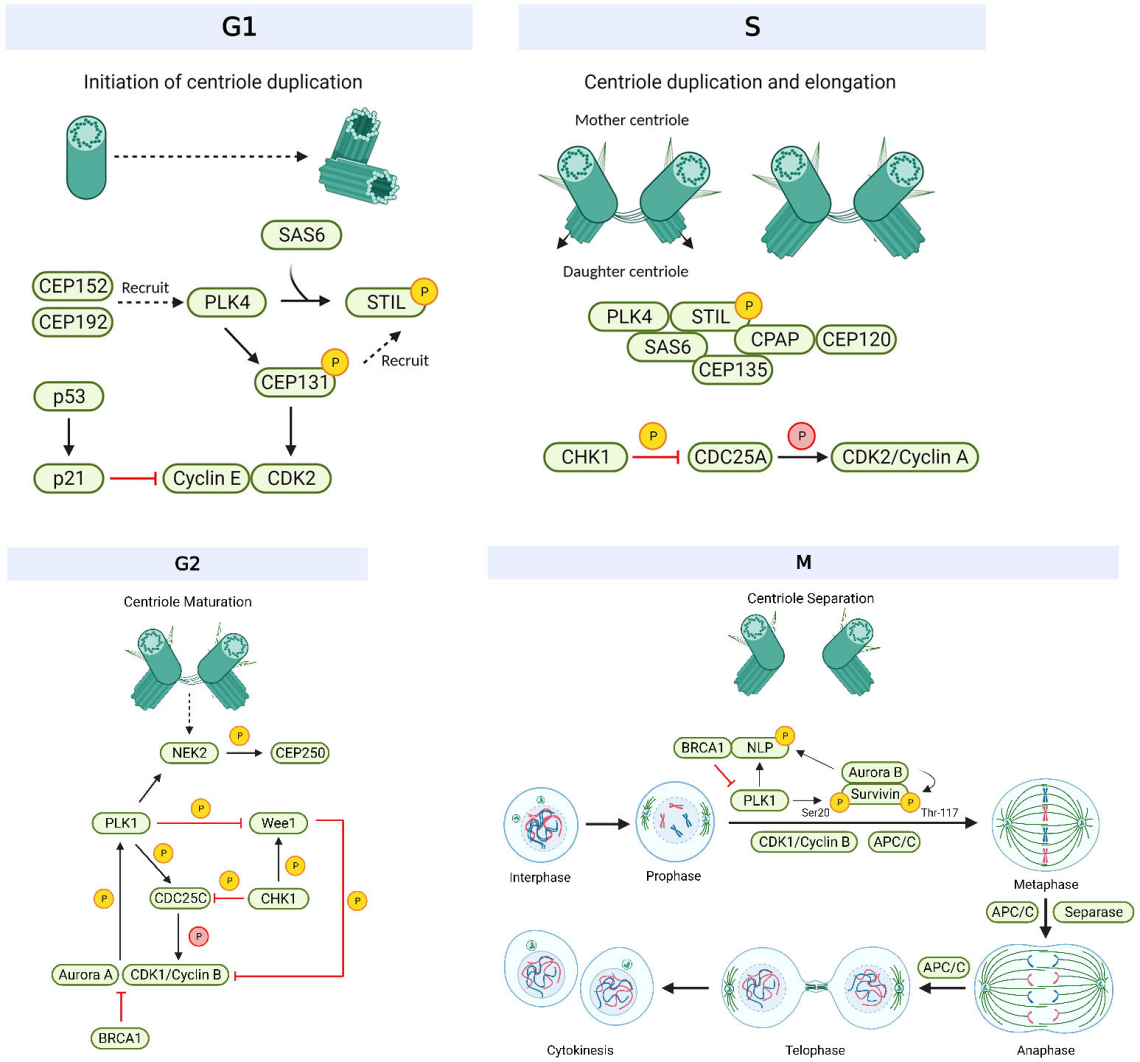
Overview of the mechanism of centrosomes during the cell cycle. Initiation of centriole duplication begins in G1. CEP152 and CEP192 recruit PLK4, which binds and phosphorylates SCL/TAL-interrupting locus protein (STIL) and associates with spindle assembly abnormal protein 6 (SAS6). STIL is recruited by CEP131, which gets phosphorylated by Polo-like kinase 4 (PLK4) and is responsible for regulating the centriole positioning of Cyclin-dependent kinase 2 (CDK2). CDK2 binds to its cyclin partner (cyclin E) but this interaction is inhibited by p21, the latter which gets activated by p53. Centriole duplication and elongation commences in S phase. The phosphorylation of STIL promotes its binding to centrosomal P4.1-associated protein (CPAP), which interacts with C-terminal encoded protein 120 (CEP120). CEP135 associates with SAS6 to link it to CPAP. Checkpoint kinase 1 (CHK1) phosphorylates and inhibits cell division cycle 25 (CDC25) A/C, which is responsible for dephosphorylating and activating CDK2/Cyclin A and CDK1/Cyclin B, respectively. CHK1 also phosphorylates and activates WEE1 during G2 (centriole maturation), which can phosphorylate and inhibit CDK1 activity. CDK1/Cyclin B interacts with Aurora kinase A to enhance its phosphorylation and activation of PLK1. Aurora kinase A activity is inhibited by BRCA1. PLK1 induces CDC25C activation and WEE1 degradation as well as regulates NIMA-related-kinase 2 (NEK2) activity. NEK2 phosphorylates CEP250 and mediates centrosome separation (M phase). Mitosis (M) is broken down into 6 steps: interphase, prophase, metaphase, anaphase, telophase, and cytokinesis. During prophase, PLK1 and Aurora kinase B phosphorylate Ninein-like protein (NLP) and survivin. The former interacts with BRCA1, and the latter activates Aurora kinase B activity. BRCA1 also inhibits PLK1 activity. CDK1/Cyclin B activates APC/C which regulates chromosome separation and degrades centrosomal components (such as SAS6, STIL, etc.) throughout mitosis. Separase regulates chromosome and centriole separation by cleaving sister chromatids during metaphase. Based on references 9, 13, 21 and 22. Created with BioRender.com.

**Figure 2:**
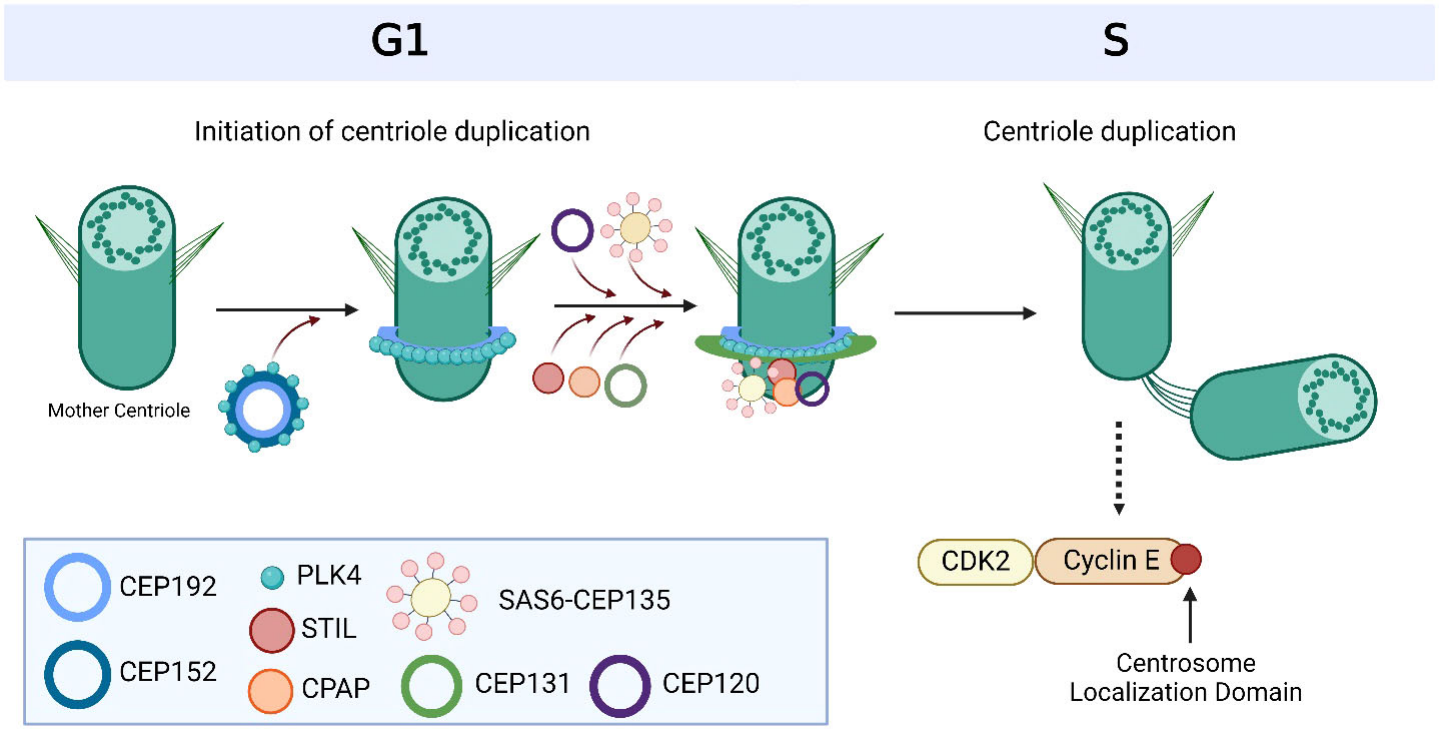
Overview of centriole duplication. To initiate centriole duplication, C-terminal encoded protein (CEP) 152 and CEP192 recruit Polo-like kinase 4 (PLK4) to the mother centriole, where it phosphorylates CEP131, which in turn recruits SCL/TAL-interrupting locus protein (STIL). PLK4 then binds and phosphorylates STIL as well as associates with spindle assembly abnormal protein 6 (SAS6) during late G1 and early S phase to initiate centriole duplication. CEP135 interacts with SAS6 to link it to centrosomal P4.1-associated protein (CPAP), which directly binds to CEP120 to induce centriole elongation of the nascent centriole. Cyclin E is recruited to the centrosome via its centrosome localization domain to promote entry into the S phase and facilitate centriole duplication. It is bound to both the centrosome and its Cyclin-dependent kinase (CDK) partner, CDK2, to facilitate centriole duplication and progression of the cell cycle. Based on references 24, 27, 150 and 212. Created with BioRender.com.

**Figure 3:**
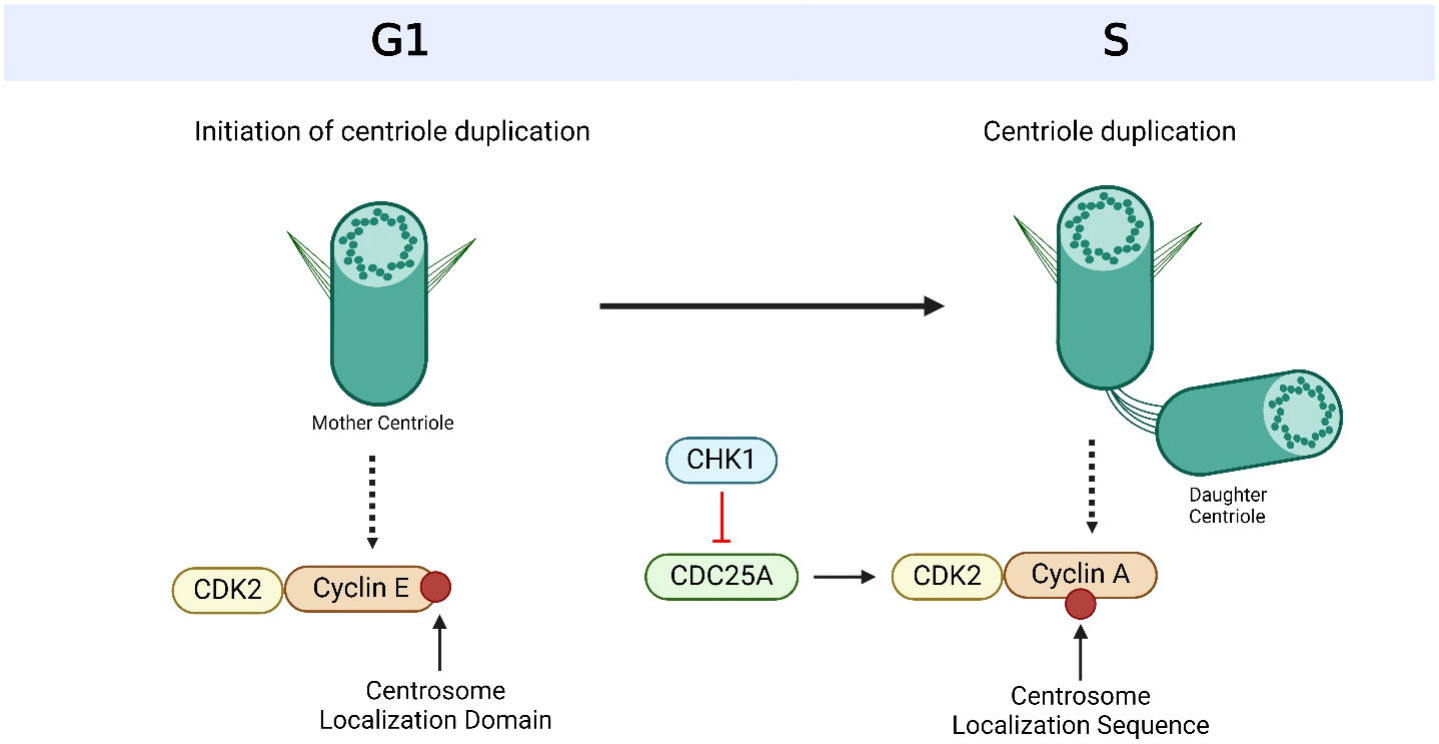
Cyclin-dependent kinases (CDKs) and their cyclin partners facilitate centriole duplication. Cyclin E is recruited to the centrosome via its centrosome localization domain and binds to the centrosome and its Cyclin-dependent kinase (CDK) partner, CDK2, to promote S phase entry and facilitate centriole duplication. Cyclin A has a centrosome localization sequence that allows it to bind to the centrosome as well as CDK2. The complex formation of cyclin A and CDK2 is promoted by cell division cycle 25A (CDC25A), which in turn is regulated by Checkpoint Kinase 1 (CHK1). Based on references 150, 151 and 238. Created with BioRender.com.

**Figure 4:**
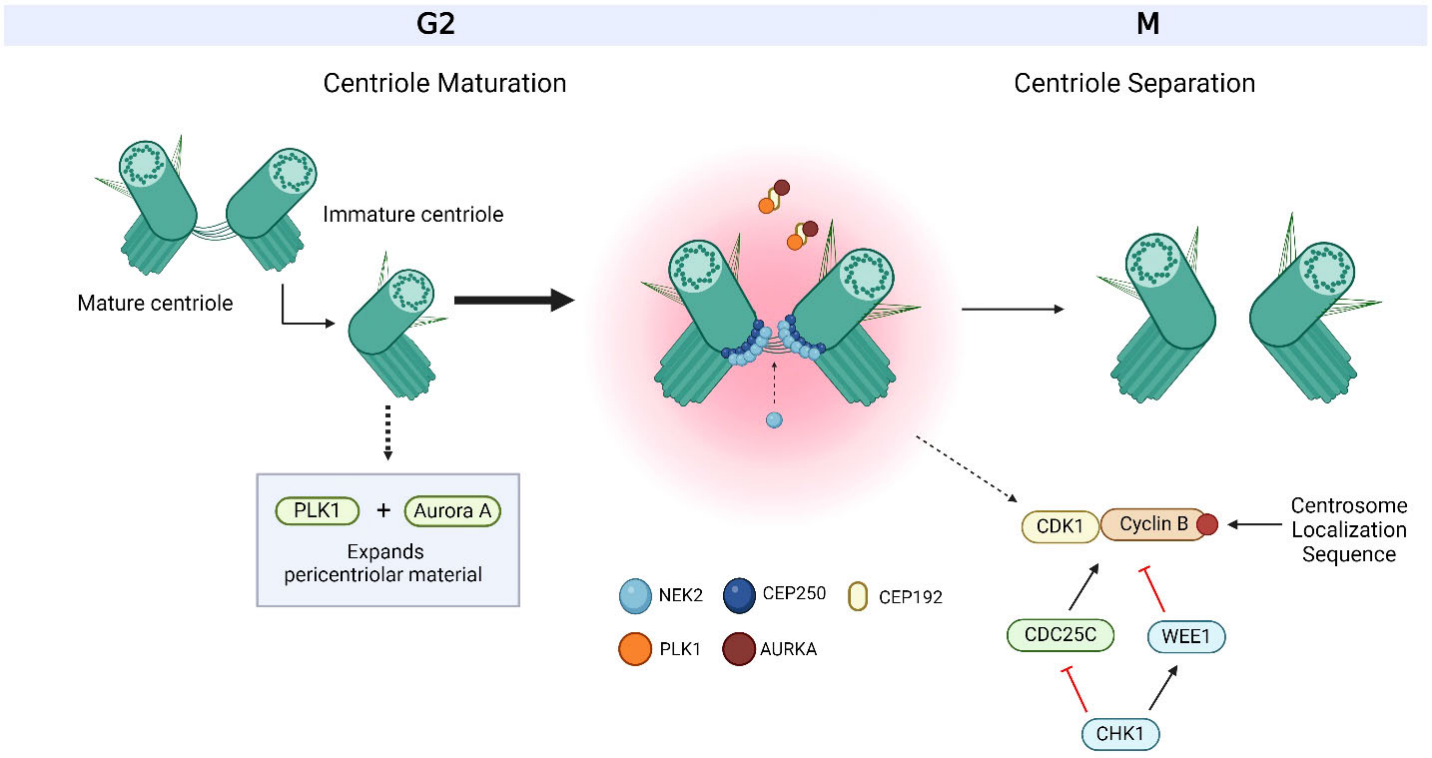
Overview of centriole maturation. Centriole maturation occurs during G2/M and is governed by both Polo-like kinase 1 (PLK1) and Aurora kinase A (AURKA). Both centrosome-associated proteins are bound and recruited to the centrosome by C-terminal encoded protein 192 (CEP192) and are responsible for expanding the pericentriolar material in order to prepare for mitotic spindle formation. CEP250 is one of the many factors that form the linkage between both centrioles and acts as a docking site for other proteins to bind to. CEP250 is bound and phosphorylated by NIMA-related-kinase 2 (NEK2) at the proximal ends of both centrioles. Together, PLK1, AURKA, CEP250 and NEK2 orchestrate centriole maturation and separation. Cyclin B is bound to the centrosome via a centrosome localization sequence at its C-terminus. The activation of cyclin B with its Cyclin-dependent kinase (CDK) partner, CDK1, occurs at the centrosome through the dephosphorylation by cell division cycle 25C (CDC25C), where its activity is regulated by Checkpoint Kinase 1 (CHK1). CHK1 also targets WEE1 for activation, which inactivates CDK1/Cyclin B. The centrosome-associated proteins, CHK1, WEE1, CDK1/Cyclin B and CDC25C also work together to facilitate centriole maturation and separation. Based on references 21, 36, 37, 152 and 234. Created with BioRender.com.

Centrosomes comprise a pair of centrioles surrounded by pericentriolar material and function as the major microtubule organizing centers in mammalian cells (17). They play an important role in regulating many cellular processes during interphase, such as cell morphology and polarization; as well as during mitosis, such as spindle formation, chromosome segregation and cytokinesis (18). These intracellular organelles are regulated by a large variety of centrosome-associated proteins throughout the cell cycle (19) (**Figure 1**, **Table 1**). The complex process of centriole duplication and maturation is orchestrated by a number of proteins during various stages of the cell cycle (**Figures 1**-**4**). During G1, centrioles prepare to divide with the help of Polo-like kinase 4 (PLK4) activity (29, 73) (**Figure 2**). Upon being recruited by centrosomal C-terminal encoded protein (CEP) 152 and CEP192 (53), PLK4 binds and phosphorylates SCL/TAL-interrupting locus protein (STIL) and associates with spindle assembly abnormal protein 6 (SAS6) to initiate centriole duplication (68, 74) (**Figure 2**). The phosphorylation of STIL promotes its binding to centrosomal P4.1-associated protein (CPAP; also known as centromere protein J [CENPJ]) (63), which interacts with CEP120 and is responsible for regulating centriole assembly, length and duplication during the S phase (46, 64) (**Figure 2**). During the G1 and S phases, Cyclin-dependent kinase (CDK) proteins also interact with their cyclin partners to facilitate centriole duplication (**Figure 3**). Centriole maturation then occurs in G2 and is primarily regulated by PLK1 and Aurora kinase A (AURKA) (**Figure 4**) (6). PLK1 regulates the localization of AURKA to the centrosome, which phosphorylates and activates PLK1 to promote bipolar spindle formation and mitotic entry (20, 31). PLK1 also regulates NIMA-related-kinase 2 (NEK2), which participates in bipolar spindle assembly to promote mitotic entry as well as mediates centrosome separation during mitosis by interacting with Centrosomal NEK2-Associated Protein 1 (C-NAP1; also known as CEP250) (21, 55, 66, 75, 76) (**Figure 4**). At this stage, the separating centrosomes form the poles of the mitotic spindle to facilitate chromosome segregation (**Figure 1**) (19).

**Table 1:** Overview of the function and aberrations of centrosome-associated proteins.

Protein	Function	Aberration
Polo-Like Kinases (PLKs)		
PLK1 (6, 20–25)	• Regulates centrosome maturation and separation, spindle assembly and mitotic entry. • Induces CDC25C activation and WEE1 degradation. • Regulates NEK2 activity and AURKA localization. • Phosphorylates and releases NLP during mitosis. • Phosphorylates and activates survivin.	• Overexpressed in breast and prostate cancer. • Overexpression leads to chromosome segregation defects, increases cancer cell proliferation, and promotes genomic instability. • Overexpression is associated with poor prognosis.
PLK2 (26, 27)	• Regulates mitosis by phosphorylating and activating proteins involved in spindle assembly, centrosome maturation, and cytokinesis, ensuring proper chromosome segregation and cell division.	• Overexpressed in colorectal cancer. • Overexpression blocked apoptotic cell death and promoted tumor growth.
PLK3 (26, 28)	• Regulates mitosis by phosphorylating and activating proteins involved in centrosome maturation, spindle formation, and chromosome alignment, ensuring proper cell division and genomic stability.	• Downregulated in colorectal cancer. • Low levels associated with worse prognosis in colorectal cancer.
PLK4 (24, 25, 29, 30)	• Centriole duplication.	• Overexpressed in breast, prostate, and cervical cancer. • Overexpression results in supernumerary centrosomes, thereby promoting genomic instability.
Aurora Kinases (AURKs)		
AURKA (6, 20, 31, 32)	• Regulates centrosome maturation and separation, spindle assembly and formation. • Phosphorylates and activates PLK1.	• Overexpressed in breast and prostate cancer. • Overexpression promotes centrosome amplification and aneuploidy.
AURKB (16, 32–34)	• Component of the chromosome passenger complex (CPC), which is essential for proper mitosis. • Controls chromosome condensation and orientation. • Phosphorylates and regulates NLP. • Regulates cell proliferation via phosphorylation of survivin.	• Overexpressed in breast and prostate cancer, NSCLC, and glioblastoma. • Overexpression leads to chromosome segregation defects, improper cell division, aneuploidy, and genomic instability.
AURKC (35)	• Role in mitosis is understudied.	• Expression is limited to testicular tissue.
Cyclin/Cyclin-Dependent Kinases (CDKs)		
CDK1/CDC2 (36–40)	• Controls the activation of CDK1/Cyclin B.	• Overexpressed in breast and colorectal cancer. • Overexpression leads to improper cell division.
CDK2 (36–40)	• Regulates mitotic entry and exit, chromosome and centrosome separation and bipolar spindle assembly. • Enhances AURKA-mediated PLK1 phosphorylation and activation. • Activates APC/C during mitosis.	• Overexpressed in breast, ovarian, pancreatic, liver, lung, thyroid, and colorectal cancer. • Overexpression leads to aberrant cell cycle progression and genomic instability.
Checkpoint Kinase 1 (CHK1)/WEE1		
CHK1 (41–43)	• DNA-damage checkpoint. • Causes temporary S and G2 phase arrest by phosphorylating and inhibiting CDC25A and C, respectively. • Phosphorylates and activates Wee1.	• Overexpressed in breast cancer (specifically TNBC), colon, and liver cancer. • Overexpression promotes cell invasion and metastasis.
WEE1 (43–45)	• DNA-damage checkpoint. • Phosphorylates and inhibits CDK1 activity, thereby causing G2 arrest.	• Overexpressed in breast, colon, and liver cancer. • Overexpression results in increased tumor cell proliferation, and subsequent genomic instability.
C-terminal Encoded Proteins (CEPs)		
CEP120 (46, 47)	• Associates with CPAP and regulates centriole elongation.	• Overexpressed in gastric cancer. • Overexpression induces the formation of excessively long centrioles.
CEP131 (48–50)	• Phosphorylated by PLK4. • Recruits STIL. • Regulates the centriole positioning of CDK2.	• Overexpressed in breast and colon cancer. • Overexpression results in supernumerary centrosomes, thereby promoting chromosomal instability, mitotic aberrations, and genomic instability.
CEP135 (51, 52)	• Participates in procentriole assembly. • Associates with SAS6 to connect it to CPAP.	• Dysregulated in breast cancer. • Dysregulation can cause defects in chromosome segregation and promote centriole amplification.
CEP152 and 192 (53, 54)	• Recruits PLK4 to centrioles.	• CEP192: Elevated levels in hepatocellular carcinoma. • Overexpression promotes cellular. proliferation and genomic instability.
CEP250 (C-NAP1) (55)	• Interacts with NEK2 to promote centrosome separation.	• Overexpressed in breast and colon cancer (TCGA).
Other		
APC/C (13, 56)	• Regulates the separation of sister chromatids and mitotic exit. • Controls the activity of CDK1/Cyclin B. • Degrades SAS6, STIL, CPAP and cyclins.	• Levels are upregulated in breast cancer. • Aberrant APC/C activity induces abnormal cellular proliferation, aneuploidy, and genomic instability.
BRCA1 (57)	• Localized at the centrosome. • Cell cycle and DNA-damage checkpoint. • Inhibits PLK1 to regulate NLP centrosome localization and protein stability. • Interacts with NLP to regulate chromosome segregation and bipolar spindle formation. • Ubiquitylates and degrades cyclin B and CDC25C. • Inhibits AURKA activity.	• Mutated or depleted in breast and ovarian cancer. • Mutation or suppression leads to improper DNA repair, aberrant cell cycle progression, aneuploidy, and genomic instability.
CDC25A (58–61)	• Dephosphorylates CDK2 rendering it active. • Regulates G1/S progression.	• Overexpressed in breast and colorectal cancer. • Overexpression promotes aberrant cycle progression, chromosome aberrations and genomic instability.
CDC25C (60, 62)	• Regulates G2/M progression and plays an important role in checkpoint protein regulation in case of DNA damage, which can ensure accurate DNA information transmission to the daughter cells. • Dephosphorylates CDK1 and activates the CDK1/Cyclin B complex.	• Overexpressed in breast, ovarian, lung, liver, gastric, bladder, prostate, and colorectal cancer. • Overexpression causes abnormal cell cycle progression, which leads to uncontrolled cell proliferation and genomic instability.
CPAP/CENPJ (63–65)	• Regulates centriole assembly, length, and duplication. • Regulates growth of microtubules during centriole assembly and elongation. • Associates with STIL.	• Overexpressed in breast cancer. • Overexpression results in unchecked cellular proliferation, which leads to genomic instability.
NEK2 (21, 66, 67)	• Role in bipolar spindle assembly. • Mediates centrosome separation. • Promotes mitotic entry. • Phosphorylates CEP250.	• Overexpressed in breast cancer. • Overexpression results in supernumerary centrosomes and multinucleated cells, thereby promoting genomic instability.
NLP (22, 33, 57)	• Regulates chromosome segregation and bipolar spindle formation. • Interacts with BRCA1. • Phosphorylated by CDK1, AURKB and PLK1.	• Overexpressed in breast, lung, and ovarian cancer. • Overexpression induces aneuploidy and genomic instability.
SAS6 (68, 69)	• Associates with STIL and PLK4.	• Overexpressed in breast and colorectal cancers. • Overexpression results in supernumerary centrosomes, thereby promoting genomic instability.
Separase (23, 70)	• Initiates anaphase. • Regulates chromosome and centriole segregation by cleaving sister chromatids.	• Overexpressed in breast cancer. • Overexpression induces aneuploidy and genomic instability.
STIL (68, 69)	• Bound and phosphorylated by PLK4.	• Overexpressed in breast, lung, and ovarian cancers. • Overexpression results in supernumerary centrosomes, thereby promoting genomic instability.
Survivin (19, 34, 71, 72)	• Localized on centrosomes, microtubules, and mitotic spindle. • Regulates chromosome cohesion and metaphase alignment. • Activates AURKB and promotes cell division.	• Overexpressed in breast, lung, gastric, colon, liver, and ovarian cancer. • Overexpression results in improper cell division, increased cancer cell proliferation and genomic instability.

In the M phase, PLK1 also phosphorylates Ninein-like protein (NLP), which interacts with BRCA1 to mediate spindle assembly and centriole separation (22, 57). NLP is also phosphorylated and regulated by AURKB (33), a major component of the chromosome passenger complex (CPC), which is responsible for correcting improper spindle attachments to the kinetochores during metaphase in order to facilitate the proper execution of mitosis (16). AURKB also controls chromosome condensation and orientation by regulating survivin, another CPC subunit (34). Survivin, which localizes to the mitotic spindle, microtubules and centrosome (19) is phosphorylated by PLK1 and activates AURKB to promote mitotic exit and complete cell division (71). During mitotic exit, separase cleaves the sister chromatids and initiates anaphase (23). Anaphase-promoting complex/cyclosome (APC/C) then facilitates the separation of the sister chromatids, thereby promoting chromosome separation (13). Chromosomes are then evenly divided into two daughter cells during cytokinesis (19). As such, centrosomes and their associated players have a crucial role throughout the cell cycle (10) and centrosome aberrations, such as defects in the structure and function of centrosomes as well as the dysregulation of their associated proteins, could lead to known hallmarks of cancer such as mitotic catastrophe and aneuploidy (17, 19, 77) (**Figure 1**).

## Centrosomes and their associated proteins in breast cancer

Centrosome aberrations are a result of the centrosome cycle being deregulated or a direct consequence of cytokinesis failure (78). This has been linked to chromosomal instability, which may support genomic changes and mutation accumulation that can encourage tumor development and resistance to therapy (79). Hence, it’s not surprising that centrosome aberrations have been implicated in various cancers and contribute to their development, growth and metastatic progression (80–83). A common centrosome aberration found in many cancers is centrosome amplification, which leads to alterations in the intricate process of cell division, thereby resulting in numerical chromosomal instability (i.e., aneuploidy or polyploidy) and the accumulation of genetic changes (84). This in turn can enhance tumorigenic properties of cancer cells. Numerical chromosomal instability induced by centrosome amplification can also accelerate the onset and spread of carcinomas via the accumulation of genetic mutations (19, 83, 85). Hence, the genomic instability that drives the development of cancer is exacerbated by these anomalies, which hinders the proper segregation of chromosomes during cell division.

Specific to this review, centrosome amplification has been strongly associated with the development and progression of breast cancer (10, 30) and numerical chromosomal instability is frequently observed in this disease (86, 87). It is a common feature of the most aggressive triple-negative breast cancer subtype, or TNBC, a highly genetically unstable disease (88). Breast tumors are fueled by this instability (89) and subsequently promote the acquisition of new mutations, which contributes to breast tumor heterogeneity (90). *In vitro* and *in vivo* studies in breast cancer have confirmed that centrosome amplification promotes tumorigenesis (10, 91, 92). However, the underlying causes of centrosome aberrations in cancer in general and in breast cancer in particular are not fully understood due to the complexities of the regulation of centrosomes and multiple proteins involved in regulating this key component of cell division. Here we highlight key proteins linked to the regulation of centrosomes and their utility as targets for therapeutic development.

## Polo-like kinases

A family of serine/threonine protein kinases known as the PLKs are involved in the control of many different cellular functions, with the regulation of centrosomes being of particular importance (26). There are five members in this family (PLK1-5) and each member has a unique function and subcellular location (26). PLK2 and PLK3 aid in regulating mitosis through centriole duplication and DNA replication respectively (26), with studies reporting tumor-promoting properties in colorectal cancer (27, 28). Meanwhile, PLK1 and PLK4 appear to be essential for centrosome regulation (93), with PLK1 controlling centrosome maturation (94) and PLK4 controlling centriole duplication (63). Hence, aberrations of PLK1 and PLK4 can cause centrosome-related errors, which in turn impact genomic stability and cell division accuracy and thereby play a vital role in cancer, including breast cancer (24, 25) (**Table 1**).

### PLK1

PLK1 is a key regulatory protein involved in cell division and preservation of genomic integrity (90). Specifically, PLK1 controls the process of centrosome maturation by phosphorylating pericentriolar material proteins to drive their expansion (**Figure 4**) (95). It was noted that in healthy cells, PLK1 expression is strictly regulated, ensuring that it is activated precisely when needed throughout G2 and mitosis (96). PLK1 has been found to be overexpressed in breast cancer (97), with up to 92% of TNBC patients showing overexpressed levels of this protein (98). Overexpression of PLK1 in breast cancer is thought to result in aberrant cell division and genomic instability (99) (**Table 1**). Higher PLK1 levels have also been linked to chemotherapy resistance (100) and aggressive tumor cell morphologies (101), making this protein an attractive biomarker candidate (102, 103) and a drug target. A number of PLK1 inhibitors, such as Rigosertib (ON-01910), Volasertib (BI 6726) and BI 2536 (104–106), have been developed (**Table 2**). These highly selective PLK1 inhibitors have been investigated in preclinical studies (104–106, 189) and have been reported to promote apoptosis, G2/M arrest and to induce anticancer effects in head and neck square cell carcinoma (Rigosertib) (105), non-small cell lung carcinoma and melanoma (Volasertib) (106), colorectal cancer (Volasertib and BI 2536) and pancreatic and cervical cancers (BI 2536) (104). BI 2536 has also been shown to reduce the invasion and metastasis of breast cancer (107). In response to BI 2536, tamoxifen-resistant MCF-7 breast cancer cells display antiproliferative effects *in vitro* and antitumor effects *in vivo (* 107). Currently, there is a lack of knowledge of the efficacy of Rigosertib and Volasertib monotherapy in breast cancer, hence clinical trials are focused on other carcinomas (115, 116, 118) (squamous cell, pancreatic, acute myeloid leukemia) and disorders (117) (myelodysplastic syndrome). However, BI 2536 has entered phase II clinical trials for patients with recurrent or metastatic solid tumors, such as breast cancer (108).

**Table 2:** Overview of drugs targeting centrosomes and associated proteins.

Drug name	Targets (IC_50_)	Effects	Preclinical Studies	Clinical Trials
Polo-like Kinase Inhibitors				
BI 2536 (104, 107, 108)	PLK1 (0.83 nM), PLK2 (3.5 nM), PLK3 (9.0 nM)	• First selective PLK1 inhibitor. • Further reduces the invasion and spread of breast cancer.	• Induced G2/M arrest and apoptotic cell death in tamoxifen-resistant MCF-7 cells and HeLa cells *in vitro.* • Inhibited tumor growth of human colon and pancreas xenografts in immunodeficient nude mice *in vivo.* • Inhibited proliferation of tamoxifen-resistant MCF-7 breast cancer cells *in vitro*. • Inhibited tumor growth and metastasis in tamoxifen-resistant MCF-7 breast cancer cells *in vivo*.	Phase II: Breast Cancer, Endometrial, Head and Neck, Melanoma, Ovarian, Sarcoma (NCT00526149)
Centrinone (109, 110)	PLK4 (2.71 nM)	• Highly selective inhibitor. • Prevents centriole formation, which blocks centriole duplication and depletes centrosomes.	• Caused p53-mediated cell cycle arrest in the G1 phase in Hela cells *in vitro.* • Blocked cell proliferation and reduced survival of MCF-7 cells *in vitro*.	N/A
Centrinone B (109, 111)	PLK4 (8.69 nM)	• Highly selective inhibitor. • Prevents centriole construction, which blocks centriole duplication and depletes centrosomes.	• Human melanoma cell lines, except those that are p53 mutant, have significantly lower cell viability and increased apoptotic cell death *in vitro*.	N/A
CFI-400945 (112–114)	PLK4 (2.8 nM)	• Oral, specific, first-in-class inhibitor. • Inhibits PLK4 by preventing its autophosphorylation at serine 305. • Induces genomic instability and aneuploidy, which results in cell cycle arrest or cell death.	• Inhibits tumor development significantly in MDA-MB-468 nude mice xenografts *in vivo.* • Anticancer effects observed in breast-, ovarian, colorectal, and lung cancer cell lines.	Phase II: TNBC (NCT03624543)
Rigosertib (ON-01910) (105, 115–117)	PLK1 (9 nM) and PLK2 (39 nM)	• Highly potent against PLK1.	• Caused cell cycle arrest and apoptosis in head and neck squamous cell carcinoma (HNSCC) *in vitro.* • Induced tumor regression in xenografts of HNSCC *in vivo.*	Phase II: Squamous cell carcinoma (NCT03786237) Phase III: Myelodysplastic syndrome (NCT01241500) and pancreatic cancer (NCT01360853)
Volasertib (BI 6727) (106, 118)	PLK1 (0.87 nM), PLK2 (5 nM), PLK3 (56 nM)	• Highly potent and selective against PLK1.	• Induced G2/M arrest and apoptosis in NSCLC cells *in vitro.* • Caused tumor regression in xenografts of neuroblastoma and colorectal cancer *in vivo*.	Phase II: Acute myeloid leukemia (NCT00804856)
YLT-11 (119)	PLK4 (22 nM)	• Causes cell death, aneuploidy, and mitotic catastrophe.	• Induced cell death in breast cancer cell lines *in vitro.* • Reduced tumor growth in human breast cancer xenograft models *in vivo*.	Phase I: Breast cancer (https://doi.org/10.1038/s41419-018-1071-2)
Aurora Kinase Inhibitors				
Alisertib (MLN8237) (120–124)	AURKA (1.2 nM)	• Highly selective inhibitor. • Second generation (predecessor to MLN8054).	• Induced cell cycle arrest, spindle aberrations, polyploidy, followed by senescence or apoptosis in multiple myeloma cells *in vitro.* • Blocked tumor growth of prostate, ovarian and colorectal cancer cell lines *in vitro*. • Caused tumor regression in neuroblastoma and lymphoma xenografts *in vivo*.	Phase II: Breast cancer (NCT01045421, NCT00807495, NCT01466881)
Barasertib (AZD1152) (125, 126)	AURKA (1.37 μM) and AURKB (0.37 nM)	• Highly selective AURKB inhibitor. • Interferes with G2, mitosis, cytokinesis.	• Inhibited growth of breast cancer cell lines *in vitro*. • Inhibited tumor growth in xenograft models of breast cancer *in vivo*.	Phase I: Advanced solid tumors (https://doi.org/10.1007/s10637-012-9825-7)
BI 811283 (120, 127, 128)	AURKB (9 nM)	• Disrupts cell cycle progression.	• Inhibited proliferation and induced polyploidy, and senescence in cancer cell lines *in vitro.* • Inhibited tumor growth of NSCLC and colorectal cancer cell line xenograft models.	Phase I: Advanced solid tumors (https://doi.org/10.1007/s00280-016-3095-6)
ENMD-2076 (129–133)	AURKA (13 nM)	• Selective against AURKA.	• Induced G2/M arrest and apoptosis in multiple myeloma cells *in vitro.* • Induced tumor regression in xenografts of breast, melanoma, and colorectal cancer.	Phase II: Ovarian cancer (NCT01104675), Triple Negative Breast Cancer (NCT01639248)
Hesperidin (134, 135)	AURKB (250 nM)	• Potent inhibitor.	• Observed antiproliferative effects in MCF7 cells *in vitro.* • Reduced tumor growth and metastasis in xenograft models of TNBC *in vivo*.	N/A
VX-680 (MK-0457) (136–140)	AURKA (0.6 nM), AURKB (18 nM), AURKC (4.6 nM)	• First inhibitor. • Reduces cell growth and induces apoptosis.	• Inhibited proliferation and induced G2/M arrest and apoptosis of clear cell renal cell carcinoma *in vitro.* • Prevents the formation of leukemia, colon, and pancreatic cancers *in vivo*.	Phase I: Cancer (NCT02532868)
VX-689 (MK-5108) (120, 141, 142)	AURKA (0.064 nM)	• Highly selective.	• Inhibited tumor growth in human tumor cell lines (breast, colon, pancreas, NSCLC) *in vitro* and in colorectal carcinoma xenograft models *in vivo*.	Phase I: Solid tumors (NCT00543387)
CDK1/2 Inhibitors				
CYC065 (Fadraciclib) (143, 144)	CDK2 (5 nM)	• Second generation.	• Induced apoptosis in trastuzumab-resistant breast cancer cells *in vitro.* • Inhibited growth of trastuzumab-resistant breast cancer xenografts *in vivo*.	Phase I: Advanced solid tumors (NCT02552953)
Dinaciclib (MK-7965; SCH 727965) (7, 145–149)	CDK1 (3 nM), CDK2 (1 nM)	• Second generation. • Therapeutic index is 10-fold higher than Flavopiridol.	• Induced apoptosis and G1 and G2/M arrest in various human tumor cell lines (breast, prostate, pancreas etc.) *in vitro.* • Inhibited cell proliferation in xenograft models of ovarian, and pancreatic cancers.	Phase II: NSCLC (10.1016/j.lungcan.2013.11.020), advanced breast cancer (10.1016/j.clbc.2013.10.016)
Flavopiridol (alvocidib) (18, 145, 150, 151)	CDK1 (30–50 nM), CDK2 (70–170 nM),	• First generation. • Suffered from low therapeutic index.	• Induced G1 and G2 arrest *in vitro*. • Induced apoptosis in mouse tissue *in vivo.* • Induced tumor regression in xenografts of lymphoma and leukemia.	N/A
HI 5 (3-hydrazonoindolin-2-one) (152)	CDK2 (1.15 μM)	• Novel anti-breast cancer drug.	• Induced G2/M arrest and apoptotic death in MCF7 cells *in vitro*.	N/A
*R*-roscovitine (seliciclib/CYC202) (18, 153)	CDK1 (650–2690 nM), CDK2 (100–710 nM)	• First generation. • Suffered from low therapeutic index.	• Induced G2/M arrest and cell death in colorectal carcinoma *in vitro.* • Growth of xenografts for colorectal and uterine cancer was mildly suppressed *in vivo*.	N/A
RGB-286638 (154, 155)	CDK1 (2 nM), CDK2 (3 nM)	• Second generation.	• Induced mitotic arrest and apoptosis and inhibited transcription in multiple myeloma cells *in vitro.* • Inhibited tumor growth and extended survival of mice bearing multiple myeloma xenografts *in vivo*.	Phase I: Advanced solid tumors (https://doi.org/10.1158/1078-0432.CCR-14-0325)
TG02 (Zotiraciclib) (156, 157)	CDK1 (9 nM), CDK2 (5 nM)	• Second generation.	• Induced G1 arrest and apoptosis in multiple cancer cell lines. (TNBC, colon, lung, melanoma) *in vitro.* • Caused tumor regression and extended survival of mice with TNBC and AML xenografts.	N/A
CHK1/WEE1 Inhibitors				
AZD1775 (MK-1775; adavosertib) (6, 158–163)	WEE1 (5.2 nM)	• Prematurely initiates mitosis, causing cell cycle arrest and consequent apoptotic cell death.	• Initiated apoptosis and cell cycle arrest in breast and colorectal cancer cells *in vitro.* • Extended survival in pancreatic, NSCLC, and glioma xenografts *in vivo*. • Observed anticancer effects in trastuzumab-resistant HER-2+ breast cancer cells.	Phase I: Advanced solid tumors (NCT0248231) Phase II: Lung, ovarian, pancreatic, stomach, and head and neck cancer
LY2606368 (prexasertib) (164, 165)	CHK1 (<1 nM), CHK2 (8 nM)	• High selectivity for CHK1.	• Caused replication catastrophe, which fragmented chromosomes and caused mitotic cell death *in vitro.* • Inhibited tumor growth in xenografts of lung cancer.	Phase II: TNBC, ovarian and prostate cancer (NCT02203513)
MK-8776 (SCH 900776) (166–169)	CHK1 (3 nM), CDK2 (160 nM)	• High potency and selectivity for CHK1.	• Induced G2/M arrest and apoptosis in cervical, lung, pancreas, and colon cancer *in vitro.* • Observed antitumor effects in breast cancer cells with p53 depletion *in vitro.* • Increased the efficacy of chemotherapy in xenograft models of pancreatic cancer *in vivo*.	Phase I: Advanced solid tumors (NCT00779584)
Others				
CCB02 (170)	CPAP-tubulin inhibitor (689 nM)	• Causes centrosome declustering, prolonged multipolar mitosis and cell death.	• Demonstrated antiproliferative activity in TNBC cells *in vitro* and in nude mice bearing human breast and lung tumor xenografts *in vivo*.	N/A
INH1 (171)	NEK2 (56 μM)	• Disrupts Hec1/NEK2 binding and triggers NEK2 degradation.	• Suppressed breast cancer cell proliferation *in vitro.* • Reduced growth of breast cancer mouse xenografts *in vivo*.	N/A
RE44 (10d) (172, 173)	CDC25A (13.5 μM) and CDC25B (4.26 μM) Inhibitor	• Disrupts CDC25A/B activity.	• Caused mouse cancer cells to enter a G2/M phase of cell cycle arrest *in vivo*. • Prevented the CDC25B substrate, CDK1 from being dephosphorylated.	N/A
Sepin-1 (174)	Separase (14.8 μM)	• Non-competitive inhibitor of separase.	• Suppressed cell proliferation and induced apoptosis. • Reduced growth of human cancer cell lines and breast cancer xenograft tumors in mice *in vivo*.	N/A
Combination Therapy				
Alisertib + Paclitaxel (175, 176)	AURKA + Microtubules	• Alisertib potentially delays the onset of acquired resistance to paclitaxel.	• Synergistic anticancer effects observed in breast cancer xenograft models *in vivo.*	Phase II: Breast cancer (NCT02187991)
AZD1775 + Paclitaxel (177–182)	WEE1 + Microtubules	• Effective against tumors with a defective G1 phase checkpoint due to loss of p53 function.	• Demonstrated antitumor activity in xenograft models of breast, ovarian and lung cancer *in vivo.*	Phase I Ovarian Cancer (NCT02272790), TNBC (NCT03012477), Gastric cancer (NCT02448329), lung cancer (NCT02513563)
CFI-400945 + Radiation (183, 184)	PLK4 + DNA damage	• Further exacerbates chromosomal instability to cause cellular lethality.	• Reduced colony formation and tumor survival in TNBC cell lines (*in vitro*) and xenograft models (*in vivo)* respectively.	N/A
Dinaciclib + Pembrolizumab (185–187)	CDK 1, CDK2 + PD-1	• Dinaciclib shown to have synergistic anticancer effects with anti-PD-1 therapy.	• Exerts synergistic anticancer effects in TNBC models.	Phase I: Advanced or metastatic breast cancer (NCT01676753) and hematological malignancies (NCT02684617)
LY2606368 + Olaparib (164, 188)	CHK1 + PARP	• LY3023414 synergizes with Olaparib to cause cell death in breast cancer cells.	• Synergistic antitumor effects observed in TNBC cell models *in vitro.*	Phase I: Advanced solid tumors (NCT03057145)

NA, Not Applicable.

### PLK4

PLK4 plays an important role in centrosome amplification in breast cancer, overseeing the essential process of centriole duplication (10, 30). By phosphorylating and activating STIL, a central part of the centriole, PLK4 allows its interaction with other centrosome-related proteins, such as SAS6 and CPAP (63) (**Figure 2**). The binding and recruitment of these other centrosome-associated proteins facilitate the process of centriole duplication (63). PLK4 is often aberrantly expressed in breast cancer, which can lead to abnormal centrosome duplication and aneuploidy, thereby inducing genomic instability and consequently contributing to breast cancer tumorigenesis and poor clinical prognosis (30). High levels of PLK4 are especially significant in TNBC, as overexpression of this gene has been observed in 48% of TNBC tumors, demonstrating the potential correlation between PLK4 and the development of TNBC (190). A more comprehensive investigation further demonstrates that PLK4 overexpression occurs in 26% of all breast cancer tumors, and this overexpression has been associated with a reduced survival rate among breast cancer patients (191). In addition to being correlated with centrosome amplification, the increased expression of PLK4 suggests that it may play a role in the development of more aggressive tumors that exhibit high-grade malignancy and a dedifferentiated cellular state (30). Understanding the relationship between centrosome amplification, PLK4 overexpression, and the poor prognosis linked to aggressive tumors, PLK4 becomes a compelling target for therapy in high-grade breast malignancies (192) (**Table 1**).

Various targeted therapeutic compounds against PLK4 have been developed, such as Centrinone, Centrinone B, CFI-400945 and YLT-11 (109, 112, 119) (**Table 2**). Centrinone and Centrinone B are highly selective PLK4 inhibitors (109). They both act by preventing centriole formation, which results in the depletion of centrosomes and subsequent cell cycle arrest (109). Centrinone has been shown to cause p53-mediated G1 arrest in cervical cancer (HeLa) cells (109), while Centrinone B induces apoptotic death in melanoma cells (111). In MCF-7 human breast cancer cells, Centrinone caused centrosome loss, which subsequently inhibited cellular proliferation and reduced survival *in vitro (* 110). However, in some cancer cells (HeLa, NIH/3T3), Centrinone treatment blocked cellular proliferation independent of centrosome loss and its effect was suggested to be insufficient for use as a single agent for cancer therapy (189). YLT-11 is another novel PLK4 inhibitor which has shown substantial antiproliferative and antitumor effects in breast cancer cells *in vitro* and *in vivo* respectively (119). Moreover, this agent has demonstrated a good safety profile with no significant toxic effects (119).

CFI-400945 emerges as the most promising candidate drug for PLK4 inhibition (**Table 2**). It is the first orally available inhibitor that targets PLK4 (112) and can also inhibit AURKB activity (113). This drug inhibits PLK4 by preventing its autophosphorylation at serine 305 (113). Upon inhibition of PLK4, it induces genomic instability and aneuploidy, eventually leading to cell death or cell cycle arrest (113). Inhibition of PLK4 has also been shown to significantly reduce tumor growth *in vivo (* 113). CFI-400945 has shown antitumor activity in preclinical studies, including TNBC, and has demonstrated to be a safe and well-tolerated drug in a phase 1 clinical trial (112, 113). It is currently being investigated in phase 2 trials (NCT03624543) in patients with TNBC (114). Although CFI-400945 is demonstrating encouraging clinical responses from monotherapy treatment, combination therapies with other DNA-damaging agents or radiotherapy which promote mitotic catastrophe may result in greater anticancer effects than each treatment alone, thereby proving to be an effective treatment strategy for breast cancer patients. Multimodality combination treatment has been investigated in TNBC models, where CFI-400945 with radiation (183, 184) has been shown to synergize anticancer effects (**Table 2**). Namely, compared to single agent treatment, the combination treatment significantly reduced colony formation in TNBC cell lines and patient-derived organoids (PDOs) *in vitro* and decreased tumor growth and improved humane endpoint survival in mouse xenografts *in vivo (* 183). Both CFI-400945 (113) and radiotherapy induce genomic instability and genotoxic stress, thus acting in combination potentially further exacerbates chromosomal instability to eventually cause cell death (192, 193).

## Aurora kinases

AURKs are a family of serine/threonine protein kinases and are vital for controlling several parts of the cell cycle, most notably mitosis (194, 195). Three members make up the AURK family (AURKA, AURKB, AURKC) (194). However, the role of AURKC in mitosis is understudied and its expression is limited to testicular tissue (35). Hence, knowledge of AURKC’s role in tumorigenesis is limited (35). Conversely, AURKA and AURKB have piqued the most interest among researchers due to their critical roles in centrosome regulation (194, 195). AURKA plays a key role in centrosome maturation and separation (16, 33, 196), specifically by phosphorylating several pericentriolar material proteins to organize the mitotic spindle (195) (**Figure 4**). While AURKB is essential for controlling chromosomal alignment and segregation during mitosis (78, 79, 197). This ensures the correct arrangement and operation of centrosomes during cell division. Overexpression of AURKA and AURKB disrupts G2 phase checkpoints (198), which results in improper chromosomal segregation during mitosis, chromosomal instability, and promotes uncontrolled cell division. These factors may contribute to tumor formation, as well as the development of more aggressive and genetically heterogeneous cancer cells (6, 199). AURKA/B have been observed to be overexpressed in various cancers, including breast (32, 200, 201). High expression of these kinases in breast cancer strongly correlates with poor survival outcomes (202). It was reported that inhibition of AURKA significantly decreased the survival of luminal and HER-2 cancer models (202). In light of this, the aberrant expression of AURKA/B in cancer emphasizes their potential value as therapeutic and drug-development targets (32, 203, 204) (**Table 1**).

Various inhibitors that target AURKA/B have been developed, such as VX-680 (MK-0457), VX-689 (MK-5108), Alisertib (MLN8237), ENMD-2076, Barasertib (AZD1152), Hesperidin, and BI 811283 (120, 134, 136) (**Table 2**). VX-680 is a first-in-class AURKA inhibitor (136). It has been shown to inhibit cellular proliferation and promote apoptotic death in various human cancers, such as ovarian and cervical cancers (136–138). It was shown to induce G2/M arrest and apoptosis in clear cell renal cell carcinoma *in vitro (* 139) as well as tumor regression in colon and pancreatic tumors *in vivo (* 140). Although initial results were encouraging, clinical trials using VX-680 were discontinued due to the concern regarding its toxicity (205). However, VX-689, a potent inhibitor of AURKA was well tolerated in preclinical models and human studies with no reports of toxic adverse effects (120). VX-689 monotherapy was shown to suppress cell proliferation in a wide range of tumors, such as breast, colon, pancreas, and NSCLC using *in vitro* models (141) and elicited anti-tumor effects in colorectal xenograft models *in vivo (* 120). A phase I clinical trial of VX-689 monotherapy in advanced solid tumor patients showed that the drug is generally well-tolerated (142). Alisertib is another highly selective and second-generation AURKA inhibitor (120, 121). Upon inhibition of AURKA, it induces G2/M arrest and aneuploidy, which subsequently leads to senescence or apoptosis in multiple myeloma cells *in vitro (* 122). Alisertib monotherapy has also been shown to inhibit tumor cell proliferation in prostate, ovarian and colorectal cancer cells *in vitro (* 121)*. In vivo* studies have shown reduced tumor growth in xenografts of neuroblastoma (123) and lymphoma (121). There are currently more than 30 clinical trials using Alisertib in a wide variety of cancers (breast, lung, ovarian, prostate) (6). In particular, there are ongoing phase II studies in breast cancer that show patient response rates of 18% when given alisertib monotherapy (124). Inhibition of AURKA by ENMD-2076 also promotes cell cycle arrest in the G2/M phase and cell death via apoptosis in multiple myeloma cells *in vitro (* 129). ENMD-2076 treatment has also been shown to diminish the growth of breast, melanoma and colorectal tumor xenografts *in vivo (* 130). Moreover, phase II trials of ENMD-2076 monotherapy in ovarian cancer have demonstrated a median overall survival of approximately 12 months, compared to 11 months without treatment (131, 132). Single-agent treatment with ENMD-2076 also resulted in clinical benefit or partial response in 16.7% of patients with TNBC in phase II trials (133), thereby highlighting its utility as a novel therapeutic for cancer patients.

AURKB inhibitors have shown mixed results in terms of their efficacy and clinical applicability (18) (**Table 2**). The selective AURKB inhibitor Barasertib showed promising results in preclinical studies in breast cancer (125) and was well tolerated in phase I trials in patients with advanced solid tumors (126). However, phase II trials were suspended after failing to show any significant clinical benefit in most patients with advanced solid tumors, including breast tumors (6). Other highly selective AURKB inhibitors include Hesperidin (134) and BI 811283 (120). Hesperidin has demonstrated antiproliferative activity in MCF7 breast cancer cells *in vitro (* 134) and anticancer effects, such as reduced tumor growth and metastasis in mouse xenograft models of TNBC *in vivo (* 135). Thus, further investigation regarding its efficacy and safety profile is warranted. BI 811283 has also shown efficacy in several solid tumors by inhibiting cell proliferation and inducing polyploidy and senescence *in vitro (* 127) and reducing tumor growth in mouse xenograft models *in vivo (* 127). Additionally, it has been demonstrated to be well-tolerated in a phase I clinical trial in patients with advanced solid tumors, including breast cancer (128).

Other novel therapeutic approaches have investigated the combination of Alisertib (175) or AZD1775 (177) with paclitaxel, a widely used anticancer agent that targets microtubules in a vast array of tumors (206) (**Table 2**). When treating breast cancer xenograft models with Alisertib and paclitaxel, synergistic or additive effects were observed (176). Moreover, in a phase II clinical study, the combination of Alisertib and paclitaxel significantly improved median progression-free survival (10.2 months) in metastatic breast cancer patients compared to either treatment alone (7.1 months) (175). This suggests that the addition of Alisertib could potentially delay the onset of acquired resistance to paclitaxel (175). Moreover, combining AZD1775 with paclitaxel in a mouse xenograft model of breast cancer resulted in the inhibition of tumor growth and extended animal survival (178). Currently, multiple clinical trials are studying this combination in various cancers harboring p53 mutations, including TNBC (179–182); the type of mutations in which AZD-1775 monotherapy has been proven to be most effective (177).

## Cyclin-dependent kinases

Another group of regulatory proteins known as CDKs is involved in centrosome control and cell cycle progression (36). There are 20 known members of the CDK family in humans, and they all play different roles in regulating different facets of cell division and proliferation (37, 38). In particular, CDK1 and CDK2 are important cell cycle regulators that play a critical role in coordinating various phases of cell division, including DNA replication and segregation (39). The activity of CDK1/2 is closely regulated by the binding of cyclins A/B (37), ensuring proper progression through the cell cycle checkpoints (201). In particular, CDK1/2 are essential for centrosome duplication and segregation through their regulatory roles in the G1/S and G2/M phases of the cell cycle (37, 207–209) (**Figures 2**-**4**, **Table 1**). Specifically in G2, CDK1 promotes centrosome maturation and separation once activated by cyclin B (210) and CDK2 phosphorylates proteins required for centriole duplication and separation (211) (**Figures 3**, **4**).

The dysregulation of CDKs can result in cell cycle aberrations and centrosome abnormalities, specifically affecting centrosome duplication and segregation, and ultimately promoting tumorigenesis (40) (**Table 1**). Studies in breast cancer have revealed elevated CDK1/2 levels in approximately 60% of tumor samples compared to normal breast tissue (32). Elevated levels of these kinases or their activating cyclins lead to disrupted cell cycle checkpoints and abnormal cell division (212). This genomic instability allows cell cycle progression even in the presence of DNA damage, enabling breast cancer cells to evade arrest and divide uncontrollably (213–215). Moreover, the overexpression of CDK1/2 interferes with DNA repair pathways, causing genomic instability and tumor growth progression (215). In HER2-positive breast tumors, overexpression of CDKs appears to promote unregulated cell cycle progression and uncontrolled tumor development (216). The control of the cell cycle in luminal breast tumors (217–219) may also be influenced by CDK overexpression, which can accelerate cell division. Consequently, targeting abnormal CDK1/2 expression and activity has emerged as a promising therapeutic strategy to suppress cancer cell proliferation and restore proper cell cycle regulation (213).

Various CDK1/2 inhibitors have been developed and investigated in preclinical and clinical studies, such as Flavopiridol (Alvocidib), R-roscovitine (Seliciclib or CYC202), Dinaciclib (MK-7965 or SCH 727965), TG02 (Zotiraciclib), CYC065 (Fadraciclib), RGB-286638 and HI-5 (18, 143, 145, 152, 154, 156) (**Table 2**). Flavopiridol and R-roscovitine are classified as first-generation pan-CDK inhibitors, which demonstrate limited specificity to various CDKs (18). Flavopiridol especially is the most extensively investigated CDK inhibitor, with over 60 clinical trials conducted between 1998 and 2014 (145). This compound effectively inhibits several CDKs by inducing G1 and G2 arrest; thereby blocking cell cycle progression and subsequently inhibiting cancer cell growth (150). These anticancer effects have been observed in breast and lung cancer cells *in vitro (* 150). Flavopiridol also reduced tumor growth in xenograft models of leukemia and lymphoma *in vivo (* 151). However, despite demonstrating strong antitumor activity in preclinical studies, phase II clinical trials with Flavopiridol alone failed to show efficacy against solid carcinomas (18). R-roscovitine also demonstrated antitumor activity by inducing cell death and G2/M arrest in colorectal carcinoma cells *in vitro* but only mildly suppressed tumor growth of colorectal and uterine carcinoma xenografts *in vivo (* 153), thereby exhibiting a lack of clinical application as a therapeutic agent. Moreover, first-generation pan-CDK inhibitors have led to relatively high rates of adverse effects (220), causing toxicities at concentrations required to effectively inhibit their targets (18).

To overcome the aforementioned drawbacks observed with first-generation CDK inhibitors, second-generation pan-CDK inhibitors have been developed (**Table 2**) (145). Dinaciclib was one of the most extensively studied second-generation pan-CDK inhibitor (145). Compared to Flavopiridol, it has a ten-fold higher therapeutic index (146) and selectively targets CDK2 with an IC50 of 1 nM (145). In preclinical studies, this compound induced cell cycle arrest in G1 and G2/M as well as initiated apoptotic cell death in multiple human cancer cell lines *in vitro*, including breast cancer (146). Additionally, dinaciclib treatment suspended tumor cell proliferation in ovarian (146) and pancreatic (147) xenograft models *in vivo.* However, early clinical trials with Dinaciclib monotherapy showed limited efficacy towards a range of solid tumors, including breast cancer (7, 148, 149). However, combining Dinaciclib with anti-PD-1 therapy showed synergistic antitumor effects in colorectal cancer compared to single-agent treatments (185). Hence, the combination of dinaciclib with PD-1 inhibitors, such as pembrolizumab has been investigated (186, 187) (**Table 2**). Dinaciclib and pembrolizumab treatment caused synergistic anticancer effects in TNBC models via attenuation of metastasis and synthetic lethality (186). Encouraging antitumor activity in hematological malignancies was also observed upon combination treatment (187). Currently, phase I trials with this combination modality are being tested in TNBC patients as well as those with leukemia, multiple myeloma and lymphoma (186, 187).

Other second-generation pan-CDK inhibitors, such as TG02, promote antiproliferative effects in a broad range of cancer cell lines (TNBC, melanoma, lung, colon) by initiating G1 arrest and apoptosis *in vitro* (**Table 2**) (156, 157). TG02 also reduced tumor growth and survival in mouse xenograft models of acute myeloid leukemia (AML) (156) and TNBC (157) *in vivo.* These results support the development of trials focused on assessing the clinical efficacy of TG02. Furthermore, CYC065 has been reported to cause apoptotic death of trastuzumab-resistant breast cancer cells *in vitro* as well as induce tumor regression in trastuzumab-resistant breast cancer xenograft models *in vivo (* 143). A phase I clinical trial in patients with advanced solid tumors also demonstrated manageable levels of toxicity upon CYC065 treatment, thereby supporting the potential of this agent as a clinical therapeutic (144). Lastly, RGB-286638 has been shown to block transcription and initiate cell cycle arrest and cell death via apoptosis in multiple myeloma cells *in vitro (* 154). Additionally, it has inhibited tumor growth and survival of multiple melanoma xenograft models *in vivo (* 154). The information regarding this compound’s efficacy in breast cancer is still lacking, although a phase I study of RGB-286638 monotherapy demonstrated to be safe and well tolerated in patients with advanced solid tumors (155). Recently, a novel and potent CDK2 inhibitor, 3-hydrazonoindolin-2-one scaffold, called HI 5 has emerged as a potential anti-breast cancer agent (152). It elicited encouraging preclinical results by demonstrating antiproliferative effects in MCF-7 breast cancer cells through the induction of growth arrest at the G2/M phase and apoptotic death *in vitro (* 152). Further studies of H1 5 are warranted to advance its development to clinical trials.

## Checkpoint Kinase 1 (CHK1)/WEE1

CHK1 and WEE1 are checkpoint kinases that control the cell cycle by slowing it down to ensure there is enough time for precise DNA replication and DNA damage repair (221). This helps preserve genomic stability and prevents the spread of potentially detrimental genetic changes (41, 221, 222). CHK1 mainly controls the G2/M checkpoint to monitor DNA damage and cell cycle progression (41), whereas WEE1 serves as a negative regulator (44) of the cell cycle by blocking CDKs (**Table 1**). In terms of their role in centrosome regulation, CHK1 localizes to interphase centrosomes and controls centrosome separation through CDK1 activity, whereas WEE1 inactivates the CDK1/Cyclin B complex through inhibitory phosphorylation to also participate in the control of centrosome separation (223) (**Figure 4**).

Overexpression of CHK1 and WEE1 has been reported in the setting of cancer (42, 43) and has been shown to aid in the growth and development of tumors (41). The overexpression of CHK1 and WEE1 in cancer cells provides them with a survival advantage over cells with normal expression levels. These proteins facilitate cancer cells to overcome genotoxic stress, promote extended cell cycle arrest, and actively assist DNA repair pathways. Thus, cancer cells are able to avoid dying and preserve the integrity of their DNA (41, 42). As a result, the normal checkpoints that prevent DNA replication errors, genomic instability, and the buildup of DNA damage are overridden by cancer cells (224). Various studies have reported overexpression of CHK1 and WEE1 in breast cancer patients (45, 225) as well as in colon and liver cancer (43) (**Table 1**). This overexpression negatively affects the sensitivity of these cells to chemicals that damage DNA in luminal breast tumors (226). Increased expression in CHK1 and WEE1 also appears to facilitate long-term survival of HER2-positive breast tumor cells (45) through the repair of DNA damage, dysregulation of the cell cycle and the evasion of apoptosis. On the other hand, inhibition of CHK1/WEE1 halts the progression of the cell cycle, causing DNA damage and premature entry of the cell into the M phase, which may trigger selective destruction of cancer cells (227).

CHK1/WEE1 are emerging as promising candidates for anticancer drug development (158, 164, 166). MK-8776, a selective and potent inhibitor of CHK1, has been shown to cause G2/M arrest and apoptotic death of cervical, lung, pancreas and colon cancer cells *in vitro (* 166). There has been a lack of research regarding its role as a monotherapy in breast cancer and *in vivo* studies. However, it has shown promising results as a radiosensitizer in TNBC models *in vitro* by inhibiting autophagy (167), as well as a chemosensitizer in pancreatic cancer xenograft models *in vivo (* 168). Phase I studies in patients with advanced solid tumor carcinomas (but not breast cancer patients) demonstrated that MK-8776 monotherapy was well tolerated (169) (**Table 2**).

LY2606368 (Prexasertib) has also demonstrated high selectivity for CHK1 (164). Treatment of cancer cells with LY2606368 triggers the activation of cell division cycle (CDC) 25A, resulting in increased levels of CDK2 (18). This drives S phase progression, which causes the accumulation of replication forks and subsequently the formation of DNA double-stranded breaks, commonly referred to as replication catastrophe (164). When studying the antitumor effects of LY2606368 in preclinical models, the compound caused the fragmentation of chromosomes and induced mitotic cell death in HeLa cells *in vitro (* 164) as well as exhibited enhanced tumor regression in a lung cancer xenograft model *in vivo (* 164). In TNBC cell lines *in vitro*, LY2606368 monotherapy caused the degradation of homologous recombination proteins (BRCA1 and RAD51) as well as induced cell cycle arrest in the S phase (228). These alterations in the homologous recombination machinery suggest the possibility of LY2606368’s utility as a combination modality with Olaparib, a well-known PARP inhibitor that targets homologous recombination deficiency in breast cancer (164). The combination of LY26006348 with Olaparib induced DNA damage and cell cycle arrest in TNBC cells *in vitro*, leading to mitotic catastrophe, genomic instability and cell death (164). A phase II clinical trial of LY26006348 monotherapy in patients with TNBC has shown limited activity (165), hence clinical trials are focused on its combination with other targeted agents, such as Olaparib (188). The combination modality of LY26006348 and Olaparib has been investigated in a phase I study in patients with metastatic solid tumors, including advanced-stage breast cancer (188) (**Table 2**).

AZD1775 (MK-1775; Adavosertib), a potent, selective and first-in-class WEE1 inhibitor, causes increased CDK1 and CDK2 levels, leading to the premature entry of cells into mitosis (158). This results in the induction of mitotic arrest and cell death via apoptosis (158). These effects were observed in colorectal cancer cells *in vitro (* 158). AZD1775 monotherapy has also been reported to extend the survival of pancreatic (159), NSCLC (160) and glioma (161) mouse xenografts *in vivo.* In HER-2 positive breast cancer, AZD1775 has shown remarkable anticancer effects by overcoming anti-HER2 agent trastuzumab resistance through the initiation of apoptosis and G2/M arrest in breast cancer cells *in vitro (* 162). These promising preclinical studies have led to numerous clinical trials of single-agent AZD1775 and its combination with chemotherapies in a variety of carcinomas (6). One, in particular, was a phase I trial of patients with advanced solid tumors such as ovarian cancer and TNBC where AZD1775 monotherapy was found to be well tolerated (163) (**Table 2**).

## C-terminal encoded proteins

The CEP family of proteins is the active component within the centrosome and consists of 31 members (48). They play a key role in cell cycle control and centriole duplication (48). In particular, CEP120, CEP131, CEP135, CEP152, CEP192 and CEP250 hold potential as therapeutic targets (48) (**Table 1**). CEP120 is known to directly interact with CPAP to elongate centrioles (**Figure 2**), and its depletion has been shown to suppress CPAP-mediated centriole elongation (46). Its overexpression is known to produce overly long centrioles, which disrupts the process of centriole duplication and promotes atypical supernumerary centrioles (46). Elevated levels of CEP120 have been observed in gastric cancers with minimal information regarding its expression in breast cancer (47). CEP131 has a vital role in the maintenance of genomic instability during cell cycle progression by regulating centriole duplication (48, 49). It does so through the phosphorylation by PLK4, which facilitates the recruitment of STIL to the centriole (50) (**Figure 2**). Upon elevated levels of CEP131, STIL is excessively recruited, which promotes supernumerary centrosomes (50). This has been observed in breast cancer, where the deubiquitinating enzyme USP9X, an integral component of the centrosome and required for centriole duplication, causes excessive levels of CEP131, thereby contributing to the pathogenesis of breast cancer (49). These findings support the promise of CEP131 as a novel target for anti-cancer interventions, particularly for breast cancer. CEP135 also plays a key role in regulating centriole duplication by interacting with SAS6 to link it to CPAP (51) (**Figure 2**). Similar to CEP120, depletion of CEP135 is known to suppress CPAP-induced centriole elongation (51), and its overexpression induces centrosome amplification (52). This has been shown to cause chromosome segregation errors in breast cancer cells, thereby promoting its carcinogenic properties (52). CEP152 and CEP192 are both involved in the recruitment of PLK4 to the centriole in order to ensure proper centriole duplication (53) (**Figure 2**). However, little is known about its tumor-promoting properties in breast cancer. A study by Liu et al. did identify CEP192 as a novel prognostic marker in hepatocellular carcinoma based on increased expression with tumor stage and association with a high mortality and recurrence rate (54). Inhibition of CEP192 blocked the proliferation of hepatocellular carcinoma cell lines, suggesting its utility as a novel anti-cancer target (54). Given these findings, more studies are warranted to determine the role of CEP152 and CEP192 in breast cancer tumorigenesis as well as its potential as a therapeutic target. Lastly, CEP250 is phosphorylated by NEK2 in order to induce centrosome separation and bipolar spindle formation (55). This is important for proper progression of the cell cycle and maintenance of genomic instability. Limited knowledge is available about the aberrant expression of CEP250 in breast cancer and cancer in general. Instead, most research has focused on studying elevated levels and inhibition of NEK2 in breast cancer tumorigenesis (229–231) (see below). Overall, there is limited knowledge about the carcinogenic mechanism of CEPs in the progression of breast cancer and more research in this field is warranted.

## Other proteins

The SAS6 and STIL proteins are vital for controlling cell division and forming the mitotic spindle, a structure that is necessary for appropriate chromosomal segregation during cell division (68). Along with PLK4, SAS6 and STIL are the core components of centriole duplication, and together they initiate the creation of a new centriole (68) (**Figure 2**). However, the development of cancer has been linked to the deregulation of these proteins (69) (**Table 1**). Changes in STIL and SAS6 levels have been linked to several subtypes of breast cancer, such as TNBC (232, 233), luminal breast cancer (234, 235), and HER2-positive breast cancer (236, 237). Tumor growth can be aided by the deregulation of these proteins, which can result in abnormal cell division and genetic instability (238, 239). By providing new paths for precision medicine in the treatment of luminal, HER2+, and TNBC subtypes, an understanding of the molecular processes involving STIL and SAS6 in breast cancer subtypes holds promise for the development of novel targeted therapeutics.

CPAP/CENPJ are essential proteins for regular cellular functions, especially those involving the control of centrosome activity and cell division (240). In order to maintain centrosome stability and appropriate chromosomal segregation, which guarantees accurate genetic material transfers to daughter cells, CPAP/CENPJ interacts with various centrosome-associated proteins (241) (**Figure 2**). These interactions allow CPAP to positively regulate centriole length and to aid in the duplication of centrosomes (63). Hence, the development of cancer may be facilitated by abnormal cell division and genomic instability brought on by disruptions in these proteins’ regular activity. A variety of cancers, including breast cancer, have been linked to overexpression in CPAP/CENPJ (65) (**Table 1**). The unchecked cell proliferation observed in luminal and HER2-positive breast cancer subtypes is thought to be associated with deregulation of centrosome activity mediated by CPAP/CENPJ (242). Moreover, abnormalities in these proteins may also affect the genomic stability of cells, which may contribute to the emergence and advancement of TNBC (243). Understanding the specific roles of CPAP/CENPJ in breast cancer subtypes provides valuable insights for the development of targeted therapeutic approaches in the future. Currently, drug treatments addressing CPAP/CENPJ are understudied regarding their use in the treatment of breast cancer. Bridging this research gap could result in new opportunities for targeted therapies and progress the field toward more tailored and successful treatments for breast cancer patients.

A family of dual-specific phosphatases known as CDC25 are essential to the control of the cell cycle (244, 245). CDC25 exists in three primary isoforms: CDC25A, CDC25B, and CDC25C (244, 245). The G1/S transition is mostly regulated by CDC25A, the G2/M transition is regulated by CDC25B and the G2/M checkpoint is dependent on CDC25C (58–60). A vital component in the proper progression of the cell cycle, including the control of centrosomes and chromosomes, is the cell cycle regulator CDC25A (61) (**Table 1**). CDC25A participates in centrosome regulation by dephosphorylating CDKs (61) (**Figure 3**). This promotes cell cycle advancement, as well as regulates centrosome duplication and maturation (61). Furthermore, CDC25A regulates apoptosis, highlighting the complexity of its role in cellular functioning (61). CDC25A dysregulation, however, has been linked to a number of malignancies, including breast cancer (58–60) (**Table 1**). CDC25A has been shown to be overexpressed in HER2-positive and TNBC subtypes (246–248). The overexpression of HER2 in HER2-positive breast tumors is correlated with overexpression of CDC25A, which may lead to unchecked cell proliferation (246). Moreover, abnormal cell cycle progression has been linked to CDC25A dysregulation in TNBC tumors (247, 248). Thus, CDC25A might serve as a potential therapeutic target in breast cancer (247). One of the compounds that has an inhibitory effect towards CDC25A/B is an o-hydroxybenzyl derivative RE44 (10d), which has shown promise in models of mouse tsFT210 breast cancer cell line *in vivo (* 172, 173) (**Table 2**).

Another key protein involved in the control of the cell cycle is CDC25C (60, 62). It typically functions as a phosphatase, helping to promote the G2 phase transition to mitosis by removing inhibitory phosphate groups from CDKs (244, 245) (**Figure 4**). It participates in centrosome regulation through the co-localization with cyclin B at the centrosomes in G2 (249) and is responsible for dephosphorylating CDK1 to activate the CDK1/Cyclin B complex to regulate centrosome maturation and separation (249). Dysregulation of CDC25C has been linked to the uncontrolled proliferation of cells (62). The fast and uncontrollable cell division observed in TNBC can be attributed to CDC25C overexpression (245, 248). Acquiring insight into CDC25C’s function in these subtypes of breast cancer could assist with developing more focused treatment strategies (**Table 1**).

A major force in controlling the progression of the cell cycle is the kinase NEK2 (250). Its typical role in mitosis consists of the maintenance of correct chromosomal segregation by aiding in the precise arrangement of centrosomes and microtubules (67). It also assists in the regulation of centrosome separation by localizing to the centrosome and controlling bipolar spindle formation as well as facilitating correct spindle attachments (67, 230). However, a number of malignancies, particularly breast cancer, have been linked to the deregulation of NEK2 (67) (**Table 1**). NEK2 overexpression has been linked to TNBC (230), luminal breast cancer (230), and HER2-positive (251) breast cancer subtypes. Increased genomic instability, tumor development, and abnormal cell cycle progression are all influenced by elevated activity of NEK2 (252). Following the inhibition of NEK2, a subsequent step involves the inhibition of CDK4/6 (253). Notably, these two therapies used together significantly reduced the extent of breast tumor in mice without causing harm to the animals *in vivo (* 253). The combination of these treatments changed the mitotic spindle genes’ mechanistic features *in vivo*, suggesting a higher degree of genomic instability (253). All these findings point to NEK2 as a potential therapeutic target for the treatment of aggressive breast malignancies like TNBC, when combined with FDA-approved CDK4/6 inhibitors. As the function of NEK2 varies in luminal, HER2-positive, and TNBC settings, knowing the precise impact of NEK2 dysregulation in distinct breast cancer subtypes can provide important insights for targeted therapy approaches. Moreover, small molecule inhibitors against NEK2, such as INH1, blocked the proliferation of several breast cancer cell lines *in vitro* as well as reduced the growth of tumor mouse xenografts of breast cancer *in vivo (* 171) (**Table 2**). Therefore, these pathways also serve as potential therapeutic strategies for breast cancer.

A crucial component of cellular machinery, APC/C controls how the cell cycle progresses. In a typical cell cycle, APC/C coordinates the degradation of certain proteins at various phases to enable appropriate cell division (254). In relation to centrosome regulation, APC/C is known to localize to the centrosome during mitosis and interact with various centrosome-associated proteins (such as CEPs) to facilitate mitotic spindle assembly (255). Dysregulation of APC/C, however, has been linked to a number of cancers, including breast cancer (56) (**Table 1**). The onset and advancement of breast cancer have been linked to aberrant APC/C activity, which affects vital cellular functions like proliferation and genomic integrity (256). For example, dysregulation of APC/C can cause unchecked cell division and genomic instability, which can aid in the development and spread of breast cancer (256). Inhibition of APC/C (13) or interference with the CPAP-tubulin interaction via CCB02 (170) prevents extra centrosomes from aggregating during mitosis, commonly referred to as centrosome clustering (**Table 2**). This helps reduce the number of cancer cells with amplified centrosomes, thereby reducing tumor growth (13, 50). These effects have been observed in TNBC and colon cancer cells *in vitro* as well as mice bearing breast and lung cancer xenografts *in vivo (* 13, 50) (**Table 2**).

Survivin, another centrosome-associated protein, is essential for controlling cell division and preventing apoptosis (257). In order to properly assemble the mitotic spindle and guarantee precise chromosomal segregation during cell division, survivin regulates centrioles and centrosomes (258). It also plays a critical function in the accurate coordination of cellular activities during mitosis by aiding in cytokinesis and stabilizing microtubules (258). Hence, abnormal survivin expression has been linked to a number of malignancies, including breast cancer (72) (**Table 1**). Survivin is frequently overexpressed in breast cancer (72) and linked to the pathogenesis of its various molecular subtypes, such as luminal (259), HER2-positive (260), and TNBC (261). Thus, there is a potential for using therapeutic targeting of survivin in breast cancer (262). However, preclinical studies of survivin inhibitors in breast cancer are limited.

Another protein that is implicated in cell division is separase, which is involved in sister chromatid separation during mitosis (263). Its regular function is to break the cohesin protein, which keeps chromatids joined together and enables precise gene transfer to daughter cells (264). During metaphase, it is known to cleave the centrosomal protein, pericentrin, allowing for centrioles to separate and be ready for the next round of centriole duplication (265). On the other hand, elevated levels of separase activity have been linked to a number of cancers, including breast cancer (70) (**Table 1**). Separase dysregulation has been connected to luminal (266), HER2-positive (267), and TNBC (268) breast cancer subtypes. The presence of hormone receptors in luminal breast tumors leads to separase hyperactivity, which can cause unchecked cell proliferation (269, 270). Separase dysregulation can render HER2+ breast tumors to be more aggressive by exacerbating the overexpression of HER2 (271). Thus, investigation of separase inhibition in breast cancer is a promising strategy. Sepin-1 has been shown to be a non-competitive inhibitor of separase, thereby preventing its enzymatic activity (174) (**Table 2**). This drug exhibits the ability to suppress cell proliferation and induce apoptosis in order to hinder the growth of human cancer cell lines and breast cancer xenograft tumors in mice (174) (**Table 2**).

## Aspects of regulation of centrosomes and their associated proteins by tumor suppressors and oncogenes in the context of breast cancer

The vastness of proteins participating in centriole duplication and maturation and regulation of centrosomes, combined with dynamic changes that take place in these processes during various phases of the cell cycle requires complex regulatory mechanisms that are provided by various cell signaling pathways, involving tumor suppressors and oncogenes, which are outside of the scope of this review. However, several such molecules are worth noting in the context of breast cancer. A guardian of the genome and a well-known tumor suppressor protein, p53, is known to participate in breast cancer pathogenesis and is found mutated in 30% of all breast tumors (272). p53 targets p21, a gene that regulates centrosome stability by inhibiting CDK2/Cyclin E during early S phase (**Figure 1**) (273). Inactivation of p53 along with the involvement of p21 leads to the induction and increase of centrosome amplification and aneuploidy in breast cancer, which is known to be associated with its poor prognosis (273). There has been emerging evidence indicating a mutual crosstalk between PLKs and p53 in cancer cells (274). For instance, PLK1 is tightly regulated by p53 and the regulation of DNA-damage repair by PLK1 is mediated through p53 among other proteins (275). However, in p53-null breast cancer cells, the regulation of PLK1 becomes dysregulated, leading to abnormal levels and in turn contributes to the development of breast cancer (275). There have also been reports of the direct interaction of AURK proteins and p53 (276). For example, the overexpression of AURKA in breast cancer may lead to enhanced degradation of p53, which in turn facilitates the development and progression of breast tumors (277).

BRCA1/2 are tumor-suppressor proteins participating in DNA damage repair pathways whose hereditary mutations are linked to the increased risk of breast cancer development (278, 279). BRCA1 is known to localize to the interphase and mitotic centrosomes and binds specifically to the mother centrioles (280). It adjusts centrosome function by inhibiting AURKA and CDK1/Cyclin B (**Figure 1**) (281). It also inhibits PLK1 in order to regulate the centrosomal localization and stability of NLP (**Figure 1**) (22). It has been reported that BRCA1 inhibition leads to centrosome amplification and aneuploidy, which promotes breast cancer tumor progression (280). It has also been shown that 62% of breast cancers overexpress AURKA, which is responsible for blocking the function of BRCA1 during G2, the phase of the cell cycle when its activity is crucial for regulating the centrosome (282). This inhibition led to the induction of supernumerary centrosomes and a more aggressive breast cancer (282).

PIK3CA is a key oncogenic protein involved in cell growth, survival and migration, and is known to be mutated in 40% of hormone receptor and HER2-positive advanced breast tumors (283). Mutations in PIK3CA lead to sustained activation of the PI3K pathway, which has been shown to induce centrosome amplification through CDK2/Cyclin E pathways (283). It was found that increased levels of PIK3CA led to higher levels of cyclin E, which in turn caused enhanced activation of centrosome duplication (284). Thereby, contributing to the overamplification of centrosomes observed in breast tumors (284).

## Discussion

Despite significant advances in diagnosis and treatment, breast cancer remains one of the leading causes of cancer-related deaths among women. It is essential to develop novel treatment approaches for the improvement of oncological outcomes in breast cancer patients. Due to abnormalities in cell division (a well-known characteristic of breast cancer cells and cancer cells in general), a focus of interest in the study of breast cancer treatment is the utilization of cell cycle proteins as therapeutic targets. In particular, targeting the centrosome and centrosome-associated proteins has piqued interest as a viable therapeutic approach due to their important role in cell cycle control.

Centrosomes are key players in the normal process of cellular division and are regulated by a large variety of centrosome-associated proteins (17, 19) (**Figure 1**, **Table 1**). The most notable proteins studied are PLKs, AURKs, CDKs, CHK1 and WEE1. These proteins have been shown to be linked to breast cancer by contributing to its development and progression, particularly through centrosome amplification, which is a common centrosome abnormality (10, 80, 84). Hence, targeting proteins involved in the regulation and function of centrosomes are attractive therapeutic targets for this aggressive disease. Currently, there are targeted therapeutics against centrosome-associated proteins in development with some in clinical trials (**Table 2**). These include the PLK4 inhibitor CFI-400945; PLK1 inhibitor BI 2536; AURKA inhibitors Alisertib and EMND-2076; AURKB inhibitor BI 811283; CHK1 inhibitor LY2606368; CDK2 inhibitor Dinaciclib and others. These agents as well as the majority of those described in this review have been shown to cause severe mitotic spindle abnormalities, such as the promotion of chromosome missegregation during mitosis in breast cancer cells. This leads to mitotic catastrophe, such as mitotic arrest and aneuploidy, which subsequently results in senescence or apoptotic cell death. Since inhibitors against these proteins have shown promising potential in preclinical models as well as clinical trials, they have strong utility to benefit breast cancer patients in the clinic.

This review synthesized a complex topic of centrosomes and their associated proteins in breast cancer, examining a vast amount of information that serves to emphasize their potential role in the future in terms of biomarker and drug discovery. While there is a number of proteins described here in the context of centrosomes and breast cancer, many of them have multiple roles that are not directly related to centrosome regulation. For example, proteins such as CHK1, WEE1 and CDKs, are known to participate in other mechanisms and pathways such as DNA-damage and DNA replication. On the other hand, some proteins have multiple roles in the complex process of centrosome regulation. For example, PLKs participate in both the maturation and duplication of centrosomes. Hence, it is difficult to discern the degree to which these proteins play a role in breast cancer through centrosome regulation and at which exact stages of this process their dysregulation participates in breast cancer pathogenesis. Furthermore, some of these proteins are not well studied in cancer, particularly in breast cancer, limiting the scope of this review. To address this, the implications of these proteins in other cancers were addressed. Lastly, the number of proteins discussed in this study are regulated by a plethora of regulatory pathways involving tumor suppressors and oncogenes, some of which have been linked to breast cancer pathogenesis. While here we address such key pathways, their detailed overview in the context of centrosomes and their regulation in breast cancer deserve a separate analysis of the literature.

Overall, this review highlights the importance of centrosomes and their associated proteins in breast cancer pathogenesis and as potential therapeutic targets for breast cancer treatment. Advances in research and drug development in this area provide encouraging results in terms of augmenting precision oncology-based treatment approaches in breast cancer required to improve outcomes in this prevalent and deadly disease. To our knowledge this is the first comprehensive review of centrosomes and their associated proteins in breast cancer, summarizing a large amount of information that could help set the stage for further scientific and clinical developments in this field.

## Author contributions

AP: Conceptualization, Writing – review & editing. HA: Conceptualization, Methodology, Supervision, Writing – original draft, Writing – review & editing. AK: Conceptualization, Writing – original draft, Writing – review & editing. VB: Conceptualization, Writing – review & editing. AA: Supervision, Writing – review & editing.
